# Unravelling and controlling hidden imprint fields in ferroelectric capacitors

**DOI:** 10.1038/srep25028

**Published:** 2016-04-28

**Authors:** Fanmao Liu, Ignasi Fina, Riccardo Bertacco, Josep Fontcuberta

**Affiliations:** 1 Institut de Ciència de Materials de Barcelona (ICMAB-CSIC), Campus UAB, Bellaterra 08193, Catalonia, Spain; 2 Institut Català de Nanociència i Nanotecnologia and The Barcelona Institute of Science and Technology (ICN2-BIST), Campus UAB, Bellaterra 08193, Catalonia, Spain; 3 LNESS Center - Dipartimento di Fisica del Politecnico di Milano, Como 22100, Italy

## Abstract

Ferroelectric materials have a spontaneous polarization that can point along energetically equivalent, opposite directions. However, when ferroelectric layers are sandwiched between different metallic electrodes, asymmetric electrostatic boundary conditions may induce the appearance of an electric field (imprint field, *E*_imp_) that breaks the degeneracy of the polarization directions, favouring one of them. This has dramatic consequences on functionality of ferroelectric-based devices such as ferroelectric memories or photodetectors. Therefore, to cancel out the *E*_imp_, ferroelectric components are commonly built using symmetric contact configuration. Indeed, in this symmetric contact configuration, when measurements are done under time-varying electric fields of relatively low frequency, an archetypical symmetric single-step switching process is observed, indicating *E*_imp_ ≈ 0. However, we report here on the discovery that when measurements are performed at high frequency, a well-defined double-step switching is observed, indicating the presence of *E*_imp_. We argue that this frequency dependence originates from short-living head-to-head or tail-to-tail ferroelectric capacitors in the device. We demonstrate that we can modulate *E*_imp_ and the life-time of head-to-head or tail-to-tail polarization configurations by adjusting the polarization screening charges by suitable illumination. These findings are of relevance to understand the effects of internal electric fields on pivotal ferroelectric properties, such as memory retention and photoresponse.

Ferroelectric materials are receiving an enormous attention due to a plethora of already real and potential applications^1^. Most of these applications are based on their excellent piezoelectric properties or on their charge storage capability, exploited in non-volatile memory applications^2^,^3^. Nowadays, flurry of research is focused on ferroelectric tunnel junctions for novel memory applications^4^,^5^,^6^,^7^,^8^,^9^ and on photo-effects in ferroelectrics for their integration in solar cells^10^,^11^,^12^,^13^. In both applications, the role of internal electric fields is very important. In the former, internal fields determine the memory retention^14^, and in the latter internal electric fields determine the magnitude and direction of the photocurrents^15^,^16^,^17^,^18^,^19^,^20^,^21^. Two types of internal electric fields can be identified: the depolarizing field (*E*_dep_) and the imprint field (*E*_imp_). The former (*E*_dep_) is opposite to the direction of the polarization, is responsible of the film depolarization and instigates breaking a single ferroelectric domain into a multi-domain structure. The latter (*E*_imp_) drives ferroelectric polarization towards a preferential direction when biasing electric fields are removed.

Imprint fields can have different origins, such as asymmetric metal-dielectric interfaces, which may give rise to unequal Schottky barriers, or graded defect concentration^22^,^23^. Consequently, to reduce *E*_imp_, electrode-ferroelectric-electrode structures should be made as symmetric as possible^24^. When dealing with ferroelectric thin films, a common approach is the use of a symmetric contact configuration in which the ferroelectric film is grown on a bottom metallic electrode and two identical top electrode pads are contacted. In this so-called, top-top measurement configuration, the two pads and the ferroelectric layer form a two capacitors series circuit connected via a common bottom electrode. As a result, symmetric polarization-vs-electric field loops can be obtained^21^,^25^. When applying an electric field (***E***) to the sample using the top-top configuration, the polarization ***P*** under each electrode points towards opposite directions. If an ***E***_**imp**_ exists across the ferroelectric layer, under one electrode ***P*** is parallel to ***E***_**imp**_ whereas under the other ***P*** is antiparallel to ***E***_**imp**_. Similarly, ***E***_**imp**_ is parallel to the applied electric field ***E*** in one capacitor but antiparallel in the other. As the switching of every ferroelectric capacitor is dictated by the actual field acting on it, their switching should occur at different ***E*** values and thus fingerprints of *E*_imp_ on *P*-*E* loops must be observable. However, as mentioned, data collected using top-top configuration typically display symmetric *P*-*E* loops, suggesting a virtual cancellation of *E*_imp_. To decipher the ultimate reasons for the absence of imprint signatures in these devices is crucial for their performance optimization and understanding.

In the present work, we study in detail the role of the *E*_imp_ on the switching process in devices formed by ferroelectric films [BaTiO_3_ (150 nm), BTO] grown on a metallic bottom electrode [La_2/3_Sr_1/3_MnO_3_ (50 nm), LSMO] using SrTiO_3_ (STO) substrates. Measurements are performed using an asymmetric contact configuration (bottom-top, sketched in Fig. 1a) and compared to those obtained in symmetric contact configuration constituted by two identical top metallic electrodes (top-top, Fig. 1b). It is found that when using top-top, no signatures of *E*_imp_ in current-field *J*-*E* and polarization-field *P*-*E* loops are observed if measurements are performed at relatively low frequencies, thus indicating the *E*_imp_ does not show up. However, when loops are recorded at higher frequency, a two-step ferroelectric switching becomes visible, which signals the presence of *E*_imp._ We argue that these results indicate that the absence of *E*_imp_ fingerprints in *P*(*E*) loops recorded using in symmetric top-top configuration, is the result of a dynamic charge polarization screening process rather than an equilibrium property of the device. Consistently, we show that single or two-step *P*-*E* loops can be obtained in a given device at a given frequency, by on-purpose modification of the screening of the polarization by using photogenerated carriers. We claim that the commonly observed symmetric *P*-*E* loops in top-top symmetric electrode configuration is the result of a *simultaneous* switching of the polarization of the ferroelectric layers of each capacitor to avoid unfavourable head-to-head/tail-to-tail domain configuration, while the *E*_imp_ is still present. These conclusions, which have been verified by experiments performed on a variety of BaTiO_3_ films grown on different substrates and using different bottom electrodes and also on BaTiO_3_ single crystals, should help to the better understanding of the response of ferroelectric-based memories and of photo-effects arising in ferroelectric materials.

## Results

### Materials

**Figure 1:**
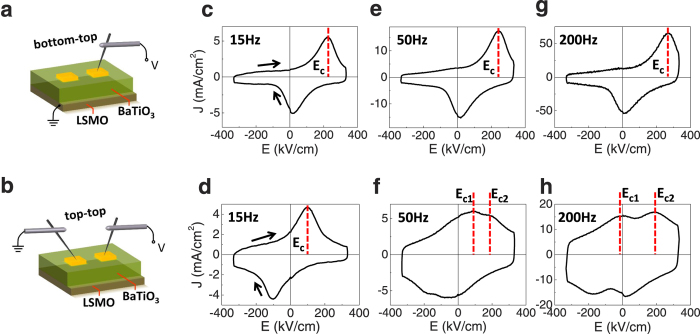
(**a**) Sketch of bottom-top (b-t) electrodes configuration for BTO (150 nm)/LSMO (50 nm)//STO sample. Top electrodes (yellow) represent the Pt top electrodes. (**b**) Sketch of top-top (t-t) electrodes configuration. (**c,e,g**) *J*-*E* loops measured in b-t at 15, 50 and 200 Hz, respectively. (**d,f,h**) *J*-*E* loops measured in t-t at 15, 50 and 200 Hz, respectively. The coercive field of single-switching-peak loops (*E*_c_) and the corresponding *E*_c1_, *E*_c2_ of double-switching-peaks loops are indicated by the (red) dashed lines. Coercive fields are only depicted for the branches of increasing applied E-field.

BaTiO_3_ (150 nm)/La_2/3_Sr_1/3_MnO_3_(50 nm) bilayers were grown by pulsed laser deposition on (001) SrTiO_3_ substrates. 20 nm thick Pt top electrodes with dimension of 60 × 60 μm^2^ and 15 μm apart (see Methods), were deposited *ex-situ* on the BTO surface by RF-sputtering. For comparison purposes, BaTiO_3_ thin films on other substrates [DyScO_3_ (DSO) and (La,Sr)(Al,Ta)O_3_ (LSAT)], or using other metallic bottom electrodes [SrRuO_3_ (SRO)] have also been tested, Consistent results have been obtained in all cases. We have also measured a BaTiO_3_ single crystal (1 mm thick). In bottom-top (b-t) configuration (Fig. 1a), the bias voltage (*V*) was applied to one of the top electrodes and the bottom electrode was grounded. In top-top (t-t) configuration (Fig. 1b), one top electrode was biased and the other was grounded. Ferroelectricity was characterized by measuring the dynamic *I*-*V* hysteresis loop to determine the switchable polarization *P* (see Methods).

### Polarization switching in symmetric and asymmetric double capacitors

Figure 1c–h show the *J*-*E* loops (arrows indicate the sense of the electric field excursion) recorded at various frequencies using b-t and t-t configurations, respectively. The corresponding *P*-*E* loops are shown in Supplementary Note 1 and Supplementary Fig. S1. In b-t configuration (Fig. 1c,e,g), it can be observed that, irrespective of the measurement frequency, the current switching peaks (indicated by dashed lines for increasing voltage) occurring at the coercive field (*E*_c_), are shifted along the positive electric field axis. This is a signature of the presence of *E*_imp_ that, in the present case, is pointing away from LSMO. A similar imprint direction has been determined by other authors on similar heterostructures^26^. From loops measured at 15 Hz we derive: *E*_imp_ = (*E*_c+ _+ *E*_c−_)/2 is ∼121.5 kV cm^−1^ and a coercive field *E*_c_ = (*E*_c+_ − *E*_c−_)/2 of about ∼100 kV cm^−1^. *E*_c±_ are the coercive fields measured at positive and negative fields, respectively. As mentioned, it is known that *E*_imp_ can originate from a variety of effects, the most obvious one being a difference of work functions of the used electrodes; in the b-t configuration: LSMO and Pt, respectively. Although other mechanisms could play a role, the important point here is that *E*_imp_ is well visible and points away from LSMO. Tests performed on other b-t contacts yield similar *E*_imp_ values. The same orientation of *E*_imp_ had also been found in similar LSMO/BTO/Pt heterostructures and it was shown to be compatible with the different of screening ability of LSMO and Pt electrodes^27^.

The results obtained using t-t contacts are radically different. Indeed, in Fig. 1d it can be appreciated that in the *J*-*E* loop measured at 15 Hz, the current switching peaks appear at symmetric electric field values thus indicating that *E*_imp_ has seemingly disappeared. This observation implies that the polarization directions of the two in-series ferroelectric capacitors have switched at the same electric field. The coercive field *E*_c_ (≈100 kV cm^−1^) is identical to that determined in the b-t configuration.

A hint to identify the underlying mechanism for the absence of *E*_imp_ in the low-frequency can be obtained by inspecting the corresponding *J*-*E* loops recorded at higher frequencies (Fig. 1f,h). At 50 Hz (Fig. 1f) the ferroelectric switching current peak becomes broader and a tiny *additional* current peak appears (both signalled by dashed lines for increasing electric field). At even higher frequency (200 Hz) the presence of two switching peaks and their splitting are more evident (Fig. 1h). This implies that the polarization switching in the two in-series capacitor device occurs as a two-step process, controlled by two different coercive fields. In brief, two switching peaks in Fig. 1f,h appear at different E-fields and their separation increases when increasing the measuring frequency. The fact that a double-switching current peak is clearly observable in the t-t but not in b-t measurements, indicates that it results from the measurement configuration rather than from an intrinsic property of the ferroelectric layer.

Similar experiments have been performed on other BaTiO_3_ thin films grown on different substrates [SrTiO_3_, DyScO_3_ and (La,Sr)(Al,Ta)O_3_] and/or using different bottom electrodes (La_2/3_Sr_1/3_MnO_3_ and SrRuO_3_). A BaTiO_3_ single crystal where different electrodes have been deposited on opposite, faces has also been tested. In all cases, consistent results have been obtained (see results of BTO/LSMO//DSO and single crystal BTO in Supplementary Note 2 and Supplementary Figs S2 and S3).

**Figure 2:**
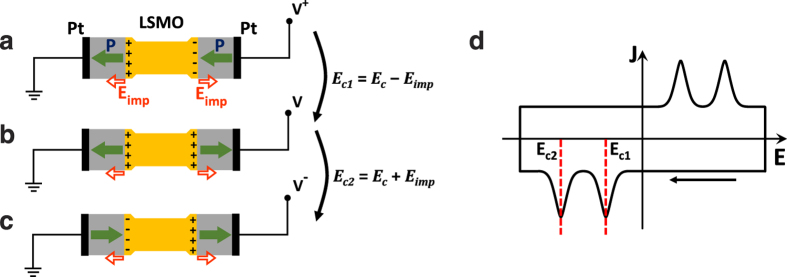
Sketches of the polarization state of the series connection of the ferroelectric capacitors of the t-t configuration, during switching in presence of *E*_imp_. Solid (green) arrows indicate the direction of *P*, and empty (red) arrows the direction of *E*_imp_. (**a**) Electric field smaller than *E*_c_ − *E*_imp_: *P* in both capacitors point towards the same direction, negative. (**b**) Electric field slightly larger than *E*_c_ − *E*_imp_: *P* in the right capacitor switches resulting in a “tail-to-tail” domain configuration. (**c**) Electric field slightly larger than *E*_c_ + *E*_imp_: the domains in the left capacitor flip, *P* in both capacitors point towards the same direction positive. (**d**) Schematic diagram of the corresponding *J*-*E* loop recorded while applying a triangular voltage pulse to the device. The pair of switching-peaks and the position of two coercive fields *E*_c1_, *E*_c2_ on the return (*V*^+^ to *V*^−^) part of the loop are marked by dashed lines.

Summarizing, when using t-t configuration, the *J*-*E* loops recorded at low frequency display a single-switching current peak whereas at high frequency, two switching peaks splits are visible. In order to understand these results we sketch in Fig. 2a–c the expected voltage-dependent polarization of two ferroelectric capacitors connected in series through a common LSMO bottom electrode. In agreement with b-t experiments (Fig. 1c,e,g), the imprint field *E*_imp_ is depicted (small empty arrow) pointing away from LSMO. We sketch the ferroelectric polarization (*P*) direction (large solid arrow) for the two ferroelectric capacitors for different values of decreasing applied electric field (from *V*^+^ to *V*^−^). In Fig. 2a, we show the situation occurring when an electric field (*V*^+^ > 0) large enough (*E*^+^ ≫ *E*_c_, *E*_imp_) to saturate the ferroelectric with the polarization pointing towards the ground, defined as negative (pointing towards the left in the figure), is applied. The polarization in both capacitors will point to the direction imposed by *E*^+^; note that the electric imprint is parallel to the polarization in one capacitor (left), but antiparallel in the other (right). When the electric field intensity reverses polarity (*E*^−^), at some *E*^−^ = *E*_c1−_ = *E*_c_ − *E*_imp_, the polarization in the right capacitor will switch; notice that│*E*_c1_│ < │*E*_c_│ because *E*_imp_ adds to help in the switching of the polarization (Fig. 2b). When the electric field is further increased (in modulus), at *E*_c2−_ = *E*_c_ + *E*_imp_ (Fig. 2c) the polarization in the left capacitor would switch as well; notice that│*E*_c2_│ > │*E*_c_│ because now *E*_imp_ is opposite to the applied electric field and thus a larger E-field is required to induce its switching. As a consequence, if imprint were to act, a “two-step” switching would occur and it will produce distinctive features at *E*_c_ − *E*_imp_ and *E*_c_ + *E*_imp_ in the *J*-*E* hysteresis loop. Thus as sketched in Fig. 2d, the *J*-*E* hysteresis loop should display two ferroelectric switching peaks. In the experimental loops of Fig. 1(f,h), these double peaks are well visible. They are superimposed to the displacive current (*i*_d_) resulting from the fact that the capacitors are charging/discharging upon voltage cycling (*i*_d_ = −d*Q*/d*t* = −d*Q*/d*V*·d*V*/d*t* = −*C*·d*V*/d*t*).

It can be observed in Fig. 1h that *E*_c1+_ = −25 kV cm^−1^, and *E*_c2+_ = +200 kV cm^−1^ at 200 Hz. These “effective” coercive field values are in good agreement with those that can be derived from the experimental *E*_c_ and *E*_imp_ determined from *J*-*E* loops recorded in b-t configuration (Fig. 1c,e,g): *E*_c1_ = *E*_c_ − *E*_imp_ = −21.5 kV cm^−1^ and *E*_c2_ = *E*_c _+ *E*_imp_ = +221.5 kV cm^−1^, confirming that the scenario depicted in Fig. 2a,b collects the physics behind. A similar analysis has been performed in other samples and coherent with results obtained. In Table 1 we collect: the *E*_imp_ and *E*_c_ values extracted from b-t and t-t configurations, respectively, at low frequency regime; the estimated values of *E*_c1_ and *E*_c2_; and experimental values of *E*_c1_ and *E*_c2_ at high frequency regime (see corresponding *J*-*E* loops in Supplementary Fig. S4). It can be observed that estimated and experimental values of *E*_c1_ and *E*_c2_ are in good agreement in samples grown on LSAT and STO irrespectively on the bottom electrode (LSMO or SRO) used. Similar good agreement is obtained for BTO/SRO//DSO sample. It is remarkable that irrespective from their slightly different structural properties (Supplementary Note 3 and Supplementary Fig. S5), the estimated and measured *E*_c1_ and *E*_c2_ values for all samples nicely agree. However, for the BTO/LSMO//DSO sample, the *E*_c_ value extracted from b-t and t-t configurations are unexpectedly different (161 and 225 kV cm^−1^, respectively). We assign this discrepancy to the fact that the properties of the bottom LSMO layer in this particular sample may be different as its anomalously shorter out-of-plane cell parameter anticipates (see Supplementary Fig. S5).

The two-step switching is signalled by the corresponding double current peaks observed in the high frequency t-t measurements; it reveals that even in the t-t configuration the *E*_imp_ is acting. However, it is not apparent in the low frequency *J*-*E* measurements. To rationalize this observation, it is worth to notice that if two-step switching process would occur, the ferroelectric capacitors would be connected head-to-head and tail-to-tail through the common bottom electrode (LSMO) as shown in Fig. 2b, which is floating, i.e. it is not grounded nor connected to a charge reservoir. This entails a poor polarization screening efficiency by the bottom electrode. Consequently, head-to-head/tail-to-tail configuration is energetically unfavourable and should revert to the stable head-to-tail/tail-to-head configurations. That is plausibly the reason why one can only indirectly observe the presence of this unfavourable domain configuration at high measurement frequencies. Switching from head-to-head (tail-to-tail) to tail-to-head (head-to-tail) occurs in the time scale of the measurement because a single current switching peak observed at low frequency. This indicates that the characteristic time for charge redistribution to screen unfavourably oriented domains is of about *τ* = 0.5/*f* ≈ 30 ms.

The *J*-*E* response in ferroelectric capacitors is dictated by the ability of the system to efficiently screen the polarization charges, and illumination of semiconducting electrodes had been used to tailor screening and the amount of switchable polarization in ferroelectric thin films^28^. Similarly, in the experiments described above, one can envisage to modulate the polarization screening by inducing photoelectrons by photon absorption at the ferroelectric layer, which in turn will modify the required screening charge density at electrodes and subsequently the relative stability of tail-to-tail and tail-to-head polarization configurations.

### Photoresponsive double capacitors

**Figure 3:**
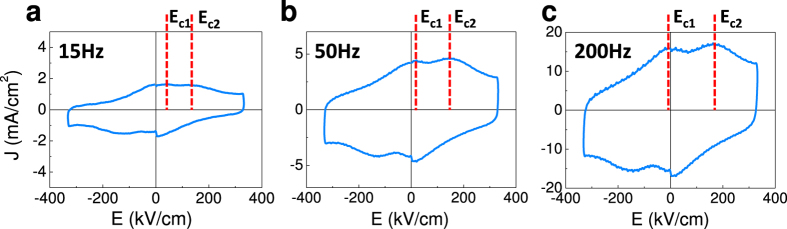
The *J*-*E* loops of sample BTO/LSMO//STO measured by t-t under illumination, at 15 Hz (**a**), 50 Hz (**b**), 200 Hz (**c**), respectively. The coercive fields *E*_c1+_, *E*_c2+_ recorded on the increasing branch of the *J*-*E* are indicated (dashed lines).

Aiming to test this proposal, we have illuminated the sample by using blue photons (3.06 eV), which it is known that can be absorbed by BaTiO_3_ thin films^29^, and we have recorded the *J*-*E* loops in t-t configuration at the same frequencies than in Fig. 1. The results are presented in Fig. 3a–c. In Fig. 3a, we show the data collected at 15 Hz. It can be observed that the magnitude of the switching current peaks measured under illumination is definitely smaller than that recorded in dark (Fig. 1). This is because photocarriers contribute to screen the polarization upon *P* reversal thus modifying the displacive current flowing in the external measuring circuit^20^. This is confirmed by the absence of any significant effect when similar experiments are performed using light of longer wavelength (see Supplementary Note 4 and Supplementary Fig. S6). Of higher relevance for the present work is the fact that in these low frequency measurements (Fig. 3a), one can observe the emergence of two current switching peaks, which were not present in the measurements performed without illumination (Fig. 1d). These two current switching peaks evolve when increasing the measuring frequency and become fully visible at 50 Hz and 200 Hz (Fig. 3b,c, respectively) whereas they were only incipient under dark conditions (Fig. 1f). These measurements indicate that under illumination, polarization switching of the two in-series ferroelectric capacitors does not proceed as a single event (within the experimental time window) but as a double-step and thus it mimics the high frequency dark response (Fig. 1d,f,h). This result is in full agreement with the picture of photocarriers modifying the electrostatic boundary conditions, namely the polarization screening, in such a way that tail-to-tail and head-to-head polar states become more stable.

**Figure 4:**
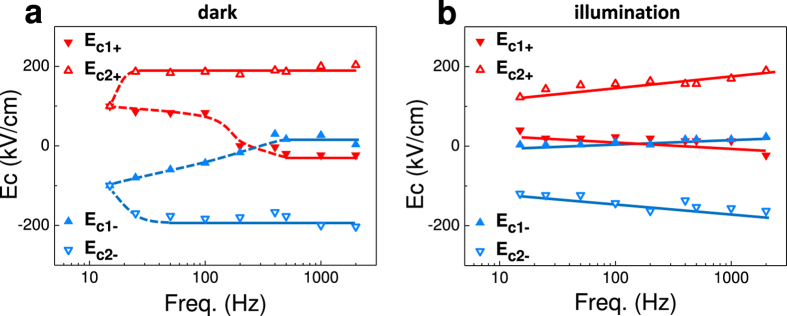
Dependence of the coercive fields on frequency, in dark (**a**) and under illumination (**b**). (*E*_c1+_, *E*_c2+_) and (*E*_c1−_, *E*_c2−_) indicate the position of the switching current peaks observed in the ascending and decreasing branches of the *J*-*E* loops, respectively.

*J*-*E* curves, similar to those show in Figs 1d,f,h and 3a–c, recorded in dark and under illumination respectively, have been measured in a range of frequencies and used to determine the frequency dependence of the coercive fields in the ascending (*E*_c1+_ and *E*_c2+_) and decreasing (*E*_c1−_ and *E*_c2−_) branches of the voltage excursion. Data are summarized in Fig. 4, where the frequency dependence of *E*_c1±_ and *E*_c2±_ is plotted. It can be appreciated that irrespectively if the sample is in dark (Fig. 4a) or under illumination (Fig. 4b), the splitting between *E*_c1+_ and *E*_c2+_ (and between *E*_c1−_ and *E*_c2−_) gradually increases when increasing frequency, indicating that faster measuring allows getting access to the unstable head-to-head and tail-to-tail configurations, having distinguishable impact on the shape of the recorded *J*-*E*. The frequency at which the two-step switching starts to be visible should be in principle limited by the electronic reordering at top or bottom electrodes, which are primarily responsible for charge screening. However, it can be appreciated in Fig. 4 that at a frequency as low as about 15 Hz, the two peaks begin to be visible. This leads to a response time *τ* ≈ 30 ms; this slow reaction time is at odds with the view that electronic transport within the electrodes determines the response time. Moreover, it is experimentally observed that the frequency dependence of the *J*(*E*) curves is independent on the distance between the t-t electrodes (either neighboring or further apart) (Supplementary Note 5 and Supplementary Fig. S7). These observations confirm that the bulk properties of the LSMO bottom electrode do not play a role. On the other hand, as the majority carrier mobility in BaTiO_3_ (typically BTO is a n-type semiconductor) is of about 1 cm^2^ V^−1^ s^−1^ and the depolarizing filed is of about 10 kV cm^−1^, the transit time for a layer of about 100 nm is expected to be much shorter (<1 ns) than the time scale (*τ*) of relevance in the present experiments. Therefore, neither carriers in the electrodes nor the majority carriers in the ferroelectric seems to determine *τ*. This conclusion is further supported by the observation that the resistance of the device decreases upon illumination (blue laser) and rapidly recovers when illumination is switched off. In contrast, although the capacitance of the device is reduced under illumination, it does not revers back to the initial state (Supplementary Note 6 and Supplementary Fig. S8), thus indicating that the change of impedance of the system is due to reordering of slow-moving charges rather than majority carriers. Therefore, minority carrier diffusion and/or ionic transport in BaTiO_3_, which are both slower processes^30^, appear to govern the observed transient response. We strength that the time constant of our measuring circuit is also much shorter (≈240 μs, see Supplementary Note 7 and Supplementary Fig. S9) and thus it cannot be relevant in the observed time response. Thus, data suggest that charge screening and concomitantly the head-to-head/tail-to-tail life-time, might be, at least partially, satisfied by electronic charge (holes) and ions in n-type BaTiO_3_.

## Conclusion

Polarization of ferroelectric thin films is often explored using contact pads placed at the film’s surface and a common metallic layer underneath is used as bottom electrode (top-top configuration). In this arrangement, the device under test is equivalent to two in-series ferroelectric capacitors connected via the common bottom electrode. Identical contacts may, *in-principle*, avoid asymmetric interface-related build-in electric fields, thus minimizing electric imprint *E*_imp_ fields. Here, we have shown that in presence of *E*_imp_, in t-t configuration, *J*-*E* loops without any signature of *E*_imp_ are only obtained when measurements are performed at relatively low frequency, whereas distinctive double-step appear when *J*-*E* is recorded at high frequency. We have shown here these effects result from the non-cancellation of imprint field, which however, only shows up at higher frequency. We have argued that the observation of double-step in *J*-*E* is a consequence of the short-living head-to-head and tail-to-tail ferroelectric domains. In other words, *E*_imp_ may not be apparent in *J*-*E* and *P*-*E* loops, but it is not cancelled in symmetric t-t configuration. We have also demonstrated that modifying the charge screening capability of the device by suitable photogenerated carrier injection, allows modulating the relative stability of head-to-head (tail-to-tail) respect to tail-to-head (head-to-tail) ferroelectric domain configuration. Finally, we have proposed that in symmetric top-top electrode measuring configuration, polarization screening is partially provided by internal charges in BaTiO_3_ which permit the camouflage of imprint fields without cancelling it. These findings may be of particular relevance in the field of photoresponse of ferroelectric films, where polarization screening plays a fundamental role.

## Methods

BaTiO_3_(150 nm)/La_2/3_Sr_1/3_MnO_3_(50 nm) bilayers were grown in a single process by pulsed laser deposition on (001) SrTiO_3_ substrates using a quadrupled Q-Switched Nd:YAG laser (*λ* = 266 nm) with a fluence of 5.2 J cm^−2^ for LSMO deposition process. For BTO growth, we used a fluence of 2.2 J cm^−2^ and a repetition rate of 2 Hz. LSMO films were grown at a deposition temperature of 730 °C and an oxygen pressure of 0.22 Torr. The growth of BTO was performed at 640 °C, with an oxygen pressure of 0.02 Torr, and it was followed by an annealing at 600 °C in a high oxygen pressure (760 Torr) during 30 min. After cooling down to room temperature, 20 nm thick Pt top electrodes were deposited on BTO surface by RF-sputtering *ex-situ*, by using a 300 mesh mask allowing to deposit about 600 electrodes (60 × 60 μm^2^, 15 μm distance apart). X-ray reciprocal space maps showed that the LSMO layer is epitaxial and fully strained whereas the pattern of BTO layer manifests an elongated out-of-plane parameter and a near relaxed in-plane cell parameter (Supplementary Note 8 and Supplementary Fig. S10). For comparison purposes, BaTiO_3_ thin films on other substrates [DyScO_3_ and (La,Sr)(Al,Ta)O_3_], using other metallic bottom electrodes (SrRuO_3_) and a BaTiO_3_ single crystal have also been tested giving consistent results.

In b-t configuration (Fig. 1a), the bias voltage (*V*) was applied to one top electrode and the bottom electrode was grounded. In t-t configuration (Fig. 1b), one top electrode was biased and the other was grounded. Unless explicitly indicated, the two electrodes used in t-t are always adjacent. Ferroelectricity was characterized by applying triangular *V*-*t* pulses of frequencies ranging from 15 Hz to 2 kHz, and measuring the dynamic *I*-*V* hysteresis loops, using a TFAnalyzer2000 (aixACCT Systems GmbH) to determine the switchable polarization *P*. The current density (*J*) is evaluated as *J* = *I*/*A* where *A* is the electrode area. The electric field is evaluated as *E* = *V*/*δ* or *V*/(2*δ*) for the b-t and t-t configurations respectively (*δ* is the ferroelectric film thickness). Further details on measurement protocols can be found elsewhere^31^.

Loops measured under illumination were collected by lighting up the electrodes with a laser of wavelength 405 nm fed by a CPX400SA DC power source (AimTTi Co.). Loops are recorded after sufficient illumination time to saturate the effect. The used photons (3.06 eV) are of sub-bandgap energy (3.3 eV for BTO^29^). It is known that oxygen deficiencies (or other point defects) in BaTiO_3_ can introduce donor states which give rise to a photoresponse under sub-bandgap illumination^32^,^33^,^34^. The spot diameter is of 200 μm, which largely covers two electrodes, allowing a homogeneous illumination with a power of 3 W cm^−2^.

## Additional Information

**How to cite this article**: Liu, F. *et al*. Unravelling and controlling hidden imprint fields in ferroelectric capacitors. *Sci. Rep.* **6**, 25028; doi: 10.1038/srep25028 (2016).

## Supplementary Material

Supplementary Information

## Figures and Tables

**Table 1:** Experimental and estimated values of *E*_c1_, *E*_c2_ of five different BTO thin film samples, grown on different substrates and using different bottom electrodes.

Sample	*E*_imp_ [kV cm^−1^]	*E*_c_ [kV cm^−1^]	Estimated [kV cm^−1^]		Experimental [kV cm^−1^]	
			*E*_c1_	*E*_c2_	*E*_c1_	*E*_c2_
BTO(150)/LSMO(50)//STO	+121.5	±100	∓21.5	±221.5	∓25	±200
BTO(90)/SRO(25)//STO	+140	±80	∓60	±220	∓18	±210
BTO(105)/LSMO(30)//DSO	+216	±219	∓3	±435	∓50	±218
BTO(90)/SRO(25)//DSO	+150	±136	∓14	±286	∓28	±328
BTO(50)/LSMO(15)//LSAT	+192	±160	∓32	±352	∓45	±388

Numbers in brackets indicate the thicknesses (nm) of the different layers. The *E*_imp_ values of b-t and *E*_c_ values of t-t are extracted from the low frequency measurements; the experimental *E*_c1_, *E*_c2_ are extracted from *J*-*E* loops recorded at high frequencies (values from loops at frequencies higher than 500 Hz, 200 Hz, 200 Hz, 1000 Hz, and 1000 Hz for sample BTO/LSMO//STO, BTO/SRO//STO, BTO/LSMO//DSO, BTO/SRO//DSO, BTO/LSMO//LSAT respectively).
