# Supplementary material for: Unravelling and controlling hidden imprint fields in ferroelectric capacitors
Source: Sci Rep. 2016 Apr 28;6:25028. doi: 10.1038/srep25028 (PMC4848548; doi:10.1038/srep25028)
Supplement: Supplementary Information [file srep25028-s1.pdf]

Supplementary Information

Unravelling and controlling hidden imprint fields in ferroelectric capacitors

Fanmao Liu, Ignasi Fina*, Riccardo Bertacco, Josep Fontcuberta

Supplementary Note 1:

In Figure S1, the *P-E* loops of BTO/LSMO//STO sample at three different frequencies recorded using b-t and t-t configurations, corresponding to the *J-E* loops in Figure 1c,e,g and Figure 1d,f,h respectively, are shown. The presence of imprint is well visible producing a noticeable shift of *P-E* loops towards positive E-field. The coercive fields do not vary appreciably with frequency. The *P-E* loops recorded in t-t configuration are symmetric and shrink dramatically as the frequency increases. The features visible in the *J-E* loops of Figure 1d,f,h, referred as double-switching peaks, are not clearly apparent in *P-E* loops because the current switching peaks are relatively small compared with the displacive current background, producing a less apparent contrast after the integration to obtain the polarization.

Supplementary Figure S1:

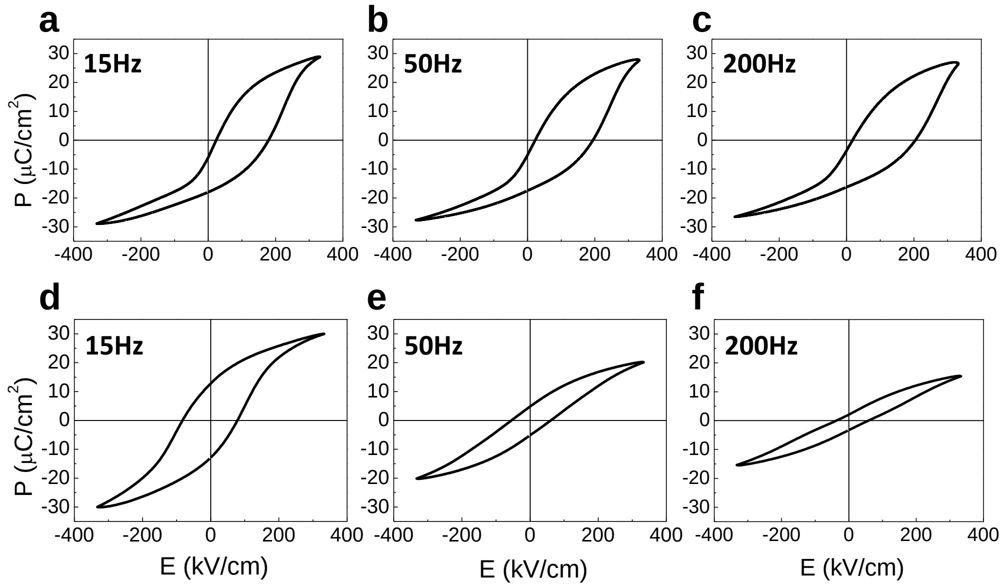

Figure S1. *P-E* loops of sample BTO/LSMO/STO measured in b-t (a,b,c) and t-t (d,e,f) configurations, at 15, 50, 200 Hz respectively, corresponding to the *J-E* loops in Figure 1c,e,g by b-t and Figure 1d,f,h by t-t, respectively.

Supplementary Note 2:

Figure S2 shows a number of *J-E* loops measured on sample $BaTiO_3$(105 nm)/$La_{2/3}Sr_{1/3}MnO_3$(30 nm)//$DyScO_3$(DSO) using the t-t configuration at various frequencies, in dark and under illumination (blue laser). Similarly, to the results reported for BTO/LSMO//STO (manuscript) double switching peaks shows up at relative high measuring frequency and they merge into one single peak at lower frequency. Under illumination, the double-switching-peaks in the *J-E* loops become more visible than in dark. They can be appreciated even at the lowest frequency, and there is a reduction of the current, particularly noticeable at the lowest frequencies. This photoresponse is analogous to that observed in BTO/LSMO//STO sample (Figure 3, the main body of the manuscript).

The $BaTiO_3$(105 nm)/$La_{2/3}Sr_{1/3}MnO_3$(30 nm) thin film bilayer was grown by pulsed laser deposition on (001) $DyScO_3$ substrates using a KrF excimer laser (λ = 248 nm) and stoichiometric targets. LSMO film was grown at 725 °C under an oxygen pressure of 0.2 mbar and a laser repetition rate of 2 Hz. The growth of BTO, performed at 700 °C and with an oxygen pressure of 0.02 mbar and 2 Hz of laser frequency. The growth rate of per laser pulse is 0.12 Å p^{-1} and 0.35 Å p^{-1} for LSMO and BTO, respectively. After cooling the samples to room temperature, top platinum electrodes (thickness 20 nm) in a 60 × 60 μm^2 dimension and 15 μm distance separating each were deposited ex-situ on the BTO surface by RF-sputtering.

A (001) cut $BaTiO_3$ single crystal (Mateck Gmbh.) with dimension 1 cm × 1 cm × 1 mm, has also been similarly characterized. Two Ag electrodes with diameter 1.5 mm were prepared by silver paste on one of the crystal (001) faces. The opposite surface was fully coated with conductive carbon paste (bottom electrode). Therefore t-t and b-t measurements can be performed, mimicking the electrodes configuration of Figure 1a,b (main body of the manuscript). The large lateral size of the sample does not allow performing measurements under illumination due to the smaller spot size of the laser.

Ferroelectric measurements on the single crystal were ran by using a similar setup as the thin film samples but by using a Trek 2205 voltage amplifier combined with the TFAnalyzer 2000, to allow reaching up to 500 V. Therefore, the maximum electric field than can be applied on the BTO single

crystal (2.5 kV cm^{-1} for t-t) is high enough to saturate its polarization. In Figure S3 we show the corresponding *J-E* data recorded in t-t at various frequencies. Obvious double switching current peaks and their variation when changing the measuring frequency are readily visible. These results are fully consistent with those recorded in BTO/LSMO//STO (Figure 1) (main body of the manuscript) and BTO/LSMO//DSO (Figure S2)

Supplementary Figure S2:

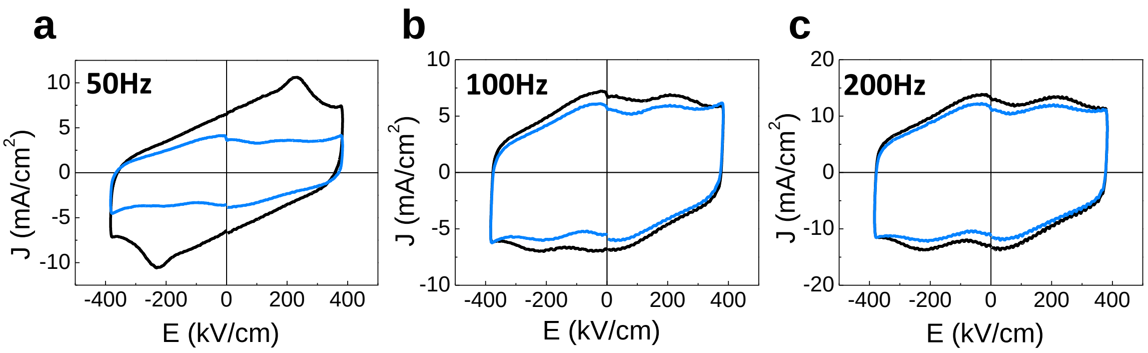

Figure S2. (a,b,c) The *J-E* loops of the sample BTO/LSMO//DSO measured in t-t configuration at 50, 100 and 200 Hz respectively, in dark (black curves) and under the illumination of a blue laser (blue curves).

Supplementary Figure S3:

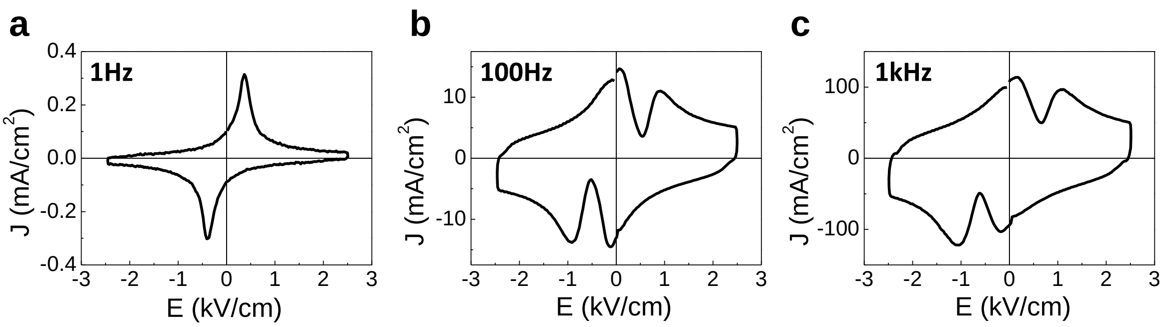

Figure S3. (a,b,c) The *J-E* loops recorded (in dark conditions) on a BTO single crystal measured in t-t configuration, at 1, 100 and 1000 Hz respectively.

Supplementary Note 3:

In Figure S4, *J-E* loops using to extracting values for Table 1 (manuscript) are shown. The four loops in the first row are of b-t measured at low frequency to extract E_{imp}, the second row shows t-t measured at low frequency to extract E_c, and the third row are t-t measured at a representative high frequency to extract experimental E_{c1}, E_{c2}.

X-ray diffraction reciprocal space maps (q-plots) of the BTO(90)/$SrRuO_3$(25)(SRO)//STO, BTO(105)/LSMO(30)//DSO, BTO(90)/SRO(25)//DSO, and BTO(50)/LSMO(15)//LSAT samples around the (103) reflection are plotted in Figure S5. Comparing the four panels in Figure S5 [and the q-plot obtained for the sample described in the manuscript (Figure S10)], it is clear that the BTO films have different in-plane and out-of-plane lattice parameters, thus implying that the films are in a different strain state and thus having partially relaxed differently. Therefore, a different microstructure is anticipated. As all these films have a similar frequency dependent $P(E)$ loops, it indicates that the phenomenology described in the manuscript is preserved irrespective from the BTO structural properties.

The $BaTiO_3$(90 nm)/$SrRuO_3$(25 nm)(SRO) and $BaTiO_3$(50 nm)/$La_{2/3}Sr_{1/3}MnO_3$(15 nm) thin film bilayers were grown by pulsed laser deposition on (001) $DyScO_3$, (001) $SrTiO_3$ and (001) $(LaAlO_3)_{0.3}(SrAl_{0.5}Ta_{0.5}O_3)_{0.7}$ substrates using a KrF excimer laser (λ = 248 nm) and stoichiometric targets. The SRO bottom electrode film was grown at 725 °C and an oxygen pressure of 0.2 mbar and 2 Hz of laser repetition rate. See Supplementary Note 2 for the growing condition of LSMO and BTO. After cooling the samples down to room temperature, 20 nm thick platinum top electrodes were deposited ex-situ on BTO surface by RF-sputtering, with dimension of 60 × 60 μm^2 and 15 μm distance separating each.

Supplementary Figure S4:

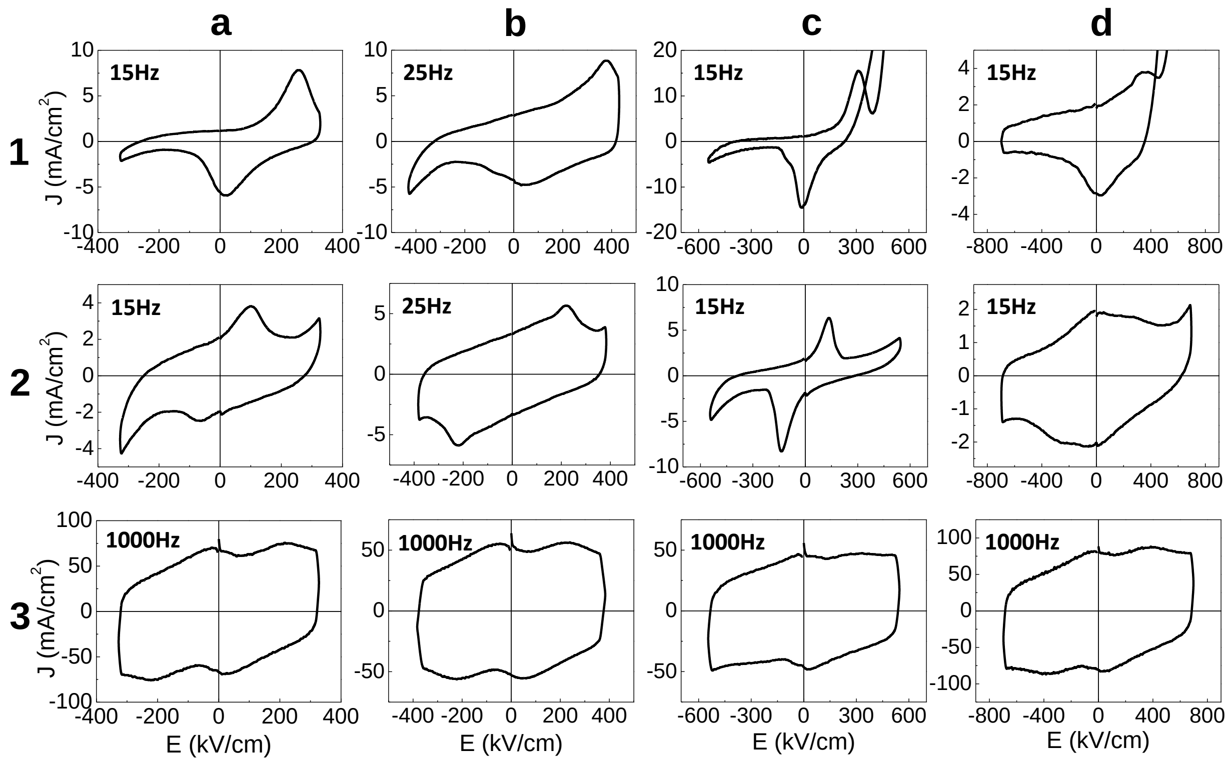

Figure S4. *J-E* loops of samples in Table 1 (except the sample BTO/LSMO//STO) recorded at representative frequencies. Column (a) BTO/SRO//STO, Column (b) BTO/LSMO//DSO, Column (c) BTO/SRO//DSO, Column (d) BTO/LSMO//LSAT. Row (1) b-t at low frequency to extract E_{imp}, Row (2) t-t at low frequency to extract E_c, Row (3) t-t at high frequency to extract experimental E_{c1}, E_{c2}.

Supplementary Figure S5:

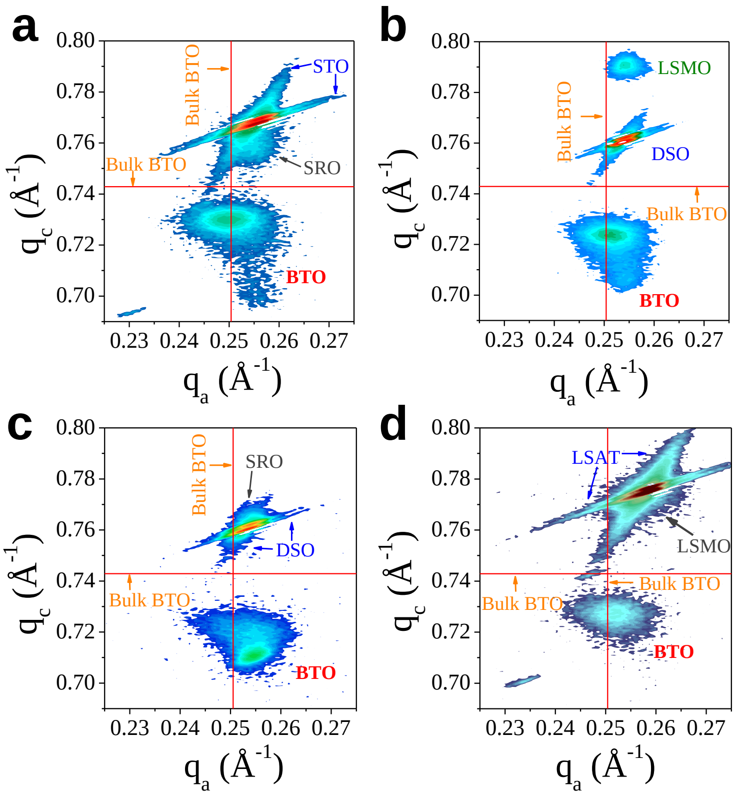

Figure S5. Reciprocal lattice maps around the (103) reflections of STO, DSO and LSAT for the (a) BTO(90)/SRO(25)//STO, (b) BTO(105)/LSMO(30)//DSO, (c) BTO(90)/SRO(25)//DSO and (d) BTO(50)/LSMO(15)//LSAT samples.

Supplementary Note 4:

In Figure S6, the *J-E* loops of the BTO/LSMO//STO sample, recorded at different frequencies in t-t configuration, under illumination with a green laser (532 nm) are compared with those recorded in dark. Measurement conditions are identical to those used in Figure 1d,f,h except for the different light wavelength. Negligible changes can be detected in the *J-E* loops, particularly at the switching peak, when comparing data under illumination (green) and in dark. This observation is consistent with the fact that the energy of the green laser (2.33 eV) is too low to activate electron-hole pairs in BTO neither across the intrinsic band gap (3.3 eV), nor at existing impurity levels. Furthermore, as the powers of the used green and blue laser are identical, the different response to different wavelengths eliminates any pyroelectric contribution affecting the observations reported here.

Supplementary Figure S6:

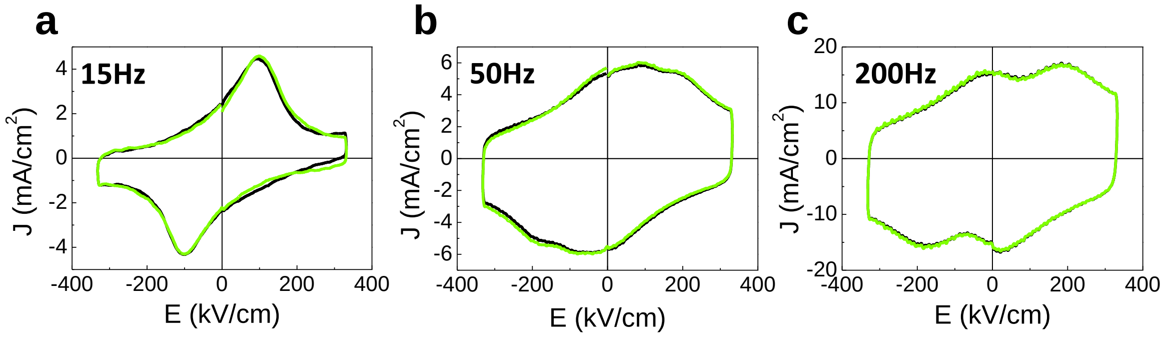

Figure S6. (a,b,c) The *J-E* loops of sample BTO/LSMO//DSO measured by t-t at 15, 50 and 200 Hz without illumination (black curves) and under the illumination of green (2.33 eV) laser (green curves).

Supplementary Note 5:

In Figure S7(b,c,d) representative *P-E* loops recorded using electrodes placed at different distances are shown (see the sketch in Figure S7a). It can be appreciated that all of them are very similar irrespective of their separation (*L*), that is: different LSMO lengths. In Figure S7(e,f,g) we show the corresponding frequency dependence of the coercive field $E_c(f)$: their close coincidence indicates that the bulk properties of the LSMO bottom electrode do not play a role.

Supplementary Figure S7:

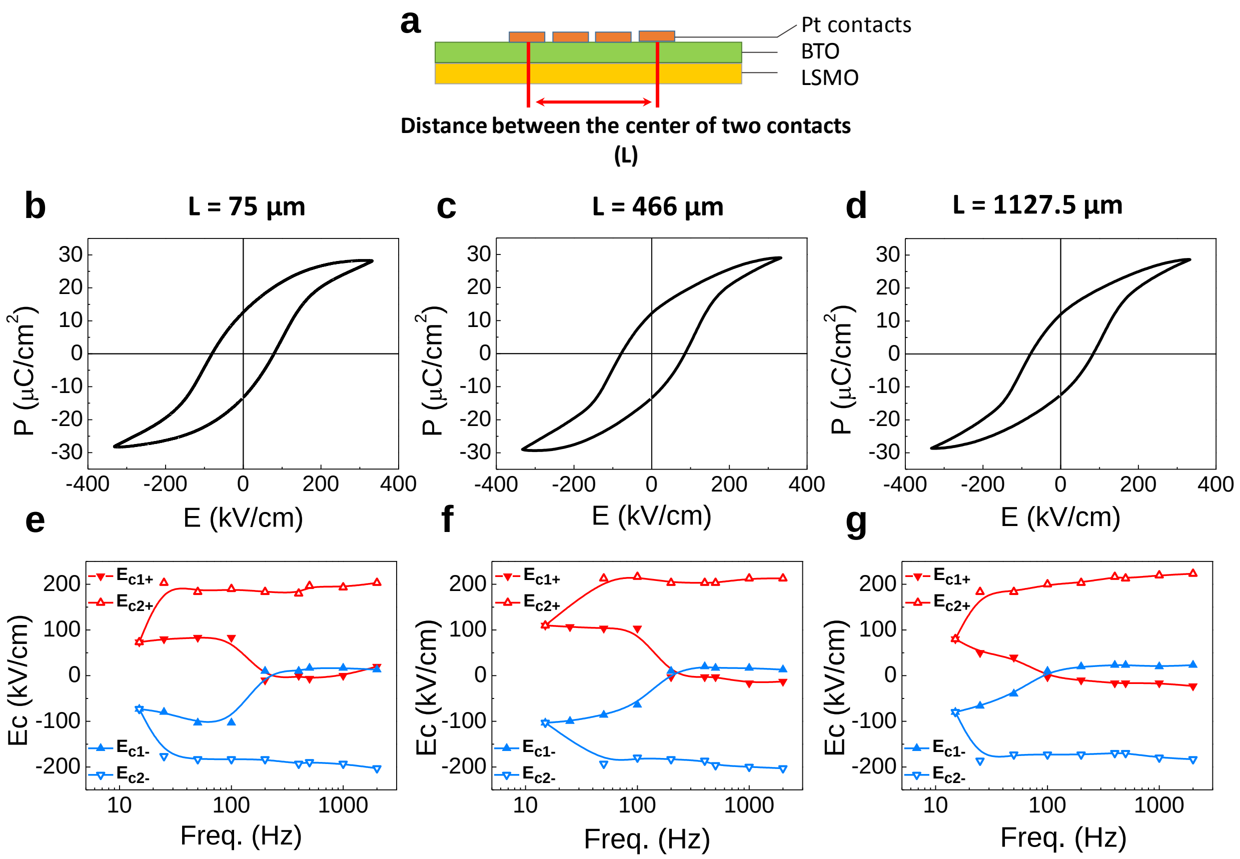

Figure S7. (a) Sketch of the t-t measurement configuration using pairs of electrodes separated different distances *L*, sample BTO/LSMO//STO (manuscript). (b,c,d) *P-E* loops recorded at 15 Hz for a pair of electrodes separated by different distances *L* as indicated. (e,f,g) Dependence on frequency of the coercive fields E_c extracted from $J(E)$measurements using pairs of electrodes separated by different distances *L*, as indicated in (b,c,d).

Supplementary Note 6:

In Figure S8a we show the *I-V* curves of BTO/LSMO//STO sample, obtained in t-t configuration in dark, during illumination (blue laser) and back to the dark state again. It can be appreciated that the resistance changes reversibly upon sample illumination. In Figure S8b, we show the capacitance of the device measured in dark (before illumination), under illumination and back to dark condition again. It can be appreciated that the capacitance is reduced by suitable illumination, but it does not recover the initial state after illumination; this implies that photo-generated carriers have produced long time living charge redistribution and impedance changes, in the device.

Supplementary Figure S8:

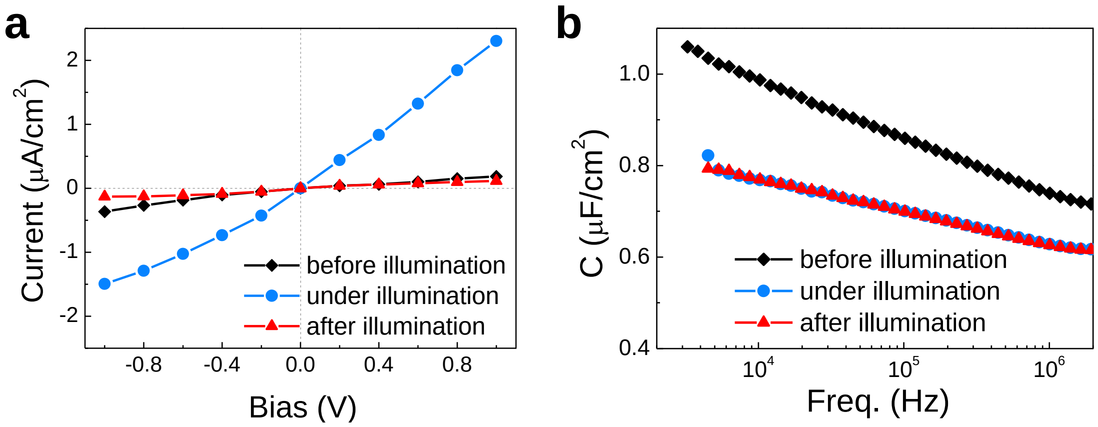

Figure S8. (a) *I*(*V*) characteristics of BTO/LSMO//STO sample measured in t-t configuration under different illumination conditions using a blue laser: before illumination (rhombi), during illumination (circle) and after illumination (triangle). (b) The device capacitance measured under the indicated illumination condition. Symbols are as in (a).

Supplementary Note 7:

In Figure S9, we shown the time dependence of the displacive current in t-t configuration, when a triangular voltage pulse is used. In (a) we depict the overall $J(t)$ during a complete measuring cycle. In (b) we display the current just after the $E(t)$ maxima. From the fitting of the $J(t) = J_0\ (1 - e^{t/\tau})$ in Figure S9(b) we extract the time constant of the circuit (τ_c). It turns out that it is of 0.24 ms ($\tau_c^{-1} \approx 4$ kHz).

Supplementary Figure S9:

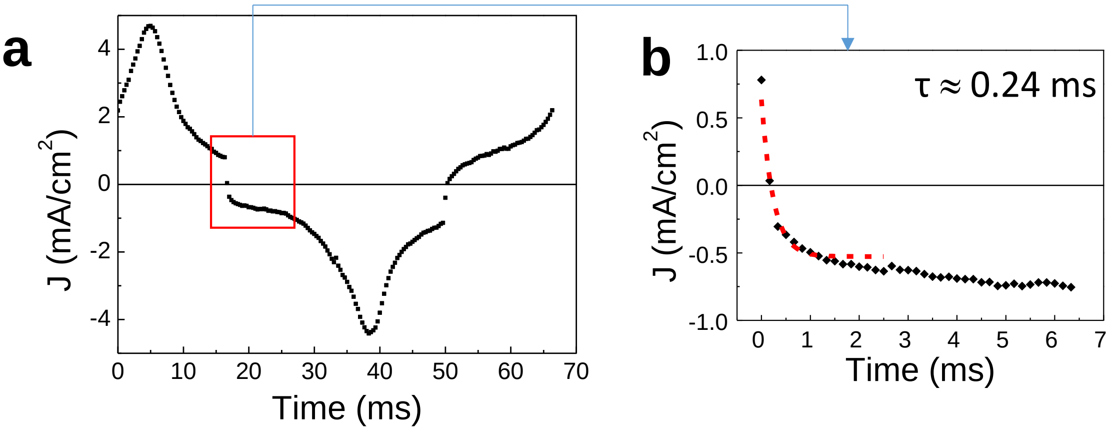

Figure S9. (a) Current dependence on time for a *P*-*E* loop recorded at 15 Hz. (b) Current vs time at the E_{max} in the $E(t)$ pulse.

Supplementary Note 8:

Figure S10 shows the X-ray diffraction reciprocal space map (q-plot) of the BTO/LSMO//STO sample around the STO (103) reflection. Data in Figure S10 reveals that the BTO film is in-plane relaxed [$a \approx 4.03(1)$ Å]. From the q-plot it can be also deduced that the c-axis lattice parameter of BTO is ~4.13(9) Å. From these values one obtains a unit cell volume of 67.25 Å^3, which is larger than its bulk value 64.38 Å^3. This difference is commonly found in BTO films and attributed to oxygen vacancies[1]. The lines indicate the position of the bulk *a,c* cell parameters (a_{bulk} = 3.992 Å, c_{bulk} = 4.036 Å).

Supplementary Figure S10:

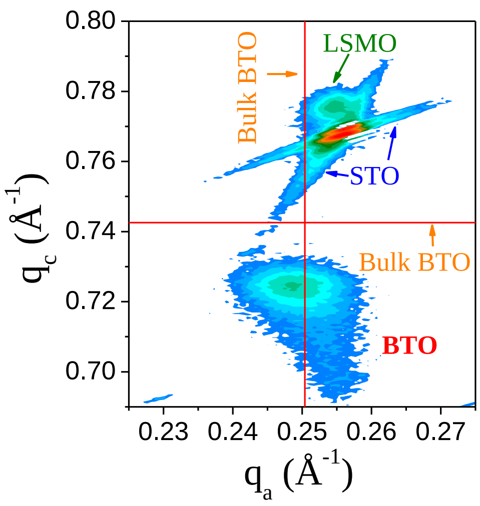

Figure S10. Reciprocal lattice maps around the (103) reflections of STO, LSMO, and BTO. The red lines indicate the position of (103) reflection of bulk BTO values (a_{bulk} = 3.992 Å, c_{bulk} = 4.036 Å).

1. Zhao, T., Chen, F., Lu, H., Yang, G., Chen, Z. Thickness and oxygen pressure dependent structural characteristics of $BaTiO_3$ thin films grown by laser molecular beam epitaxy. *J. Appl. Phys.* **87,** 7442–7447 (2000).
